# Nicotine self-administration and ERK signaling are altered in RasGRF2 knockout mice

**DOI:** 10.3389/fphar.2022.986566

**Published:** 2022-09-02

**Authors:** Ilaria Morella, Veronika Pohořalá, Claudia Calpe-López, Riccardo Brambilla, Rainer Spanagel, Rick E. Bernardi

**Affiliations:** ^1^ Neuroscience and Mental Health Innovation Institute, Cardiff University, Cardiff, United Kingdom; ^2^ Division of Neuroscience, School of Biosciences, Cardiff University, Cardiff, United Kingdom; ^3^ Institute of Psychopharmacology, Central Institute of Mental Health, Medical Faculty Mannheim, University of Heidelberg, Mannheim, Germany

**Keywords:** RasGRF2, nicotine, self-administration (SA), pERK, extracellar signal-regulated kinase, pERK1/2

## Abstract

Ras/Raf/MEK/ERK (Ras-ERK) signaling has been demonstrated to play a role in the effects of drugs of abuse such as cocaine and alcohol, but has not been extensively examined in nicotine-related reward behaviors. We examined the role of Ras Guanine Nucleotide Releasing Factor 2 (RasGRF2), an upstream mediator of the Ras-ERK signaling pathway, on nicotine self-administration (SA) in RasGRF2 KO and WT mice. We first demonstrated that acute nicotine exposure (0.4 mg/kg) resulted in an increase in phosphorylated ERK1/2 (pERK1/2) in the striatum, consistent with previous reports. We also demonstrated that increases in pERK1/2 resulting from acute (0.4 mg/kg) and repeated (0.4 mg/kg, 10 daily injections) exposure to nicotine in WT mice were not present in RasGRF2 KO mice, confirming that RasGRF2 at least partly regulates the activity of the Ras-ERK signaling pathway following nicotine exposure. We then performed intravenous nicotine SA (0.03 mg/kg/infusion for 10 days) in RasGRF2 KO and WT mice. Consistent with a previous report using cocaine SA, RasGRF2 KO mice demonstrated an increase in nicotine SA relative to WT controls. These findings suggest a role for RasGRF2 in the reinforcing effects of nicotine, and implicate the Ras-ERK signaling pathway as a common mediator of the response to drugs of abuse.

## Introduction

Worldwide tobacco smoking rates have been on the decline for several years, due in large part to the MPOWER measures instituted by the World Health Organization (World Health Organization, 2021). Nevertheless, tobacco remains one of the leading causes of preventable deaths worldwide, with an estimated 8 million smoking-related deaths annually (World Health Organization, 2021). Nicotine is the primary reinforcing component of tobacco, and continued tobacco use can result in nicotine dependence in humans. Particularly worrisome is the dramatic increase in the use of electronic nicotine delivery systems (ENDS) or e-cigarettes, especially among adolescents, which may lead to the use of traditional tobacco products (Leventhal et al., 2015; Ren and Lotfipour, 2019; Park et al., 2020). In addition to the potential development of nicotine dependence, the use of ENDS likely have health consequences similar to those described for traditional tobacco products, such as cardiovascular and respiratory diseases (Glynos et al., 2018; Davidson and Fox, 2020; Raja et al., 2021). Thus, continuing to identify and understand the molecular mechanisms that underlie nicotine reinforcement is critical to the development of strategies to further counteract tobacco use and pharmacotherapies for nicotine dependence.

The extracellular signal-regulated kinases (ERK) cascade (Ras/Raf/MEK/ERK; Ras-ERK), a member of the mitogen-activated protein kinase (MAPK) family, is an important signaling pathway linking signals from cell surface receptors with changes in gene expression (Grewal et al., 1999; Orton et al., 2005). This pathway has been implicated in the acute and long-term effects of drugs of abuse, including cocaine and other psychostimulants (Valjent et al., 2006; Papale et al., 2016; Bernardi et al., 2019), alcohol (Sanna et al., 2002; Faccidomo et al., 2009; Agoglia et al., 2015; Faccidomo et al., 2015), and nicotine (Brunzell et al., 2003; Valjent et al., 2004; Valjent et al., 2005; Wilar et al., 2019), likely regulating striatal drug-dependent synaptic plasticity (Girault et al., 2007; Cerovic et al., 2013). In terms of nicotine, administration (0.4 mg/kg) in mice has been demonstrated to increase the phosphorylation of ERK1/2 (pERK1/2) in the ventral and dorsal striatum (Valjent et al., 2004; Valjent et al., 2005; but see Brunzell et al., 2003), mesolimbic dopamine structures that have both been shown to mediate neuroadaptations associated with drug dependence (Koob and Volkow, 2010; Lobo and Nestler, 2011). In addition, studies of nicotine conditioned place preference in mice have demonstrated the potential involvement of ERK in nicotine reward, as nicotine conditioning increased pERK1/2 relative to saline conditioning in the nucleus accumbens (NAc) (Wilar et al., 2019; Jia et al., 2021). And self-administration studies have also demonstrated the involvement of ERK and downstream targets of ERK in nicotine reinforcement (Konu et al., 2001; Li et al., 2012; Gomez et al., 2015). Thus, ERK is clearly involved in the molecular and behavior effects of nicotine; however, less is known about the role of upstream regulators of ERK that may mediate these effects.

Ras guanine nucleotide exchange factors (GEFs) are critical mediators of the activity of the Ras-ERK signaling pathway. For example, Ras Guanine Nucleotide Releasing Factor 2 (RasGRF2) is one of several GEFs that mediates the cycling of the Ras family of small GTPases from an inactive GDP-bound state to an active GTP-bound state in a Ca2/calmodulin-dependent way via various neurotransmitter receptors (Zippel et al., 1997; Quilliam et al., 2002; Pamonsinlapatham et al., 2009; Feig, 2011), allowing the transduction of extracellular signals to downstream effectors such as MEK and ERK (Takai et al., 2001; Feig, 2011). Thus, given the role of the Ras-ERK cascade in drug-mediated plasticity, RasGRF2 likely functions as a critical mediator of reinforcement processes that precipitate drug dependence.

We recently demonstrated that cocaine self-administration (SA) was increased in RasGRF2 knock-out (KO) mice relative to their wild-type littermates (WT), which we attributed to a decrease in cocaine reinforcement and compensatory increase in intake to achieve similar putative reward (Bernardi et al., 2019). Importantly, these alterations in cocaine intake were accompanied by an attenuation of the cocaine-induced increase in pERK in the ventral and dorsal striatum in RasGRF2 KO mice. Furthermore, these molecular and behavioral findings in RasGRF2 KO mice were replicated in C57Bl/6 mice using the MEK inhibitor PD325901. These findings suggest that RasGRF2 mediates the cellular response to cocaine via Ras-ERK signaling (Bernardi et al., 2019). RasGRF2 has also been demonstrated to mediate alcohol reinforcement, as RasGRF2 KO mice showed a reduction in alcohol drinking and an attenuation of the increase in dopamine induced by alcohol in the nucleus accumbens (NAc) (Stacey et al., 2012). Thus, RasGRF2 likely plays a general role in reward processes by mediating the activity of the Ras-ERK pathway in striatal regions (Mazzucchelli and Brambilla, 2000; Stornetta and Zhu, 2011).

The goal of the current set of experiments was to examine the role of RasGRF2 in nicotine reinforcement using nicotine self-administration in a separate cohort of RasGRF2 KO and WT mice. We first sought to demonstrate a role for ERK resulting from acute nicotine administration in male C57Bl/6N mice by performing immunohistochemistry for pERK1/2 in the striatum. We also performed pERK1/2 immunohistochemistry in RasGRF2 KO and WT male mice in these regions following both acute and repeated nicotine administration. We then performed nicotine SA in RasGRF2 KO mice and WT littermate controls; here we used both male and female mice to identify potential sex differences in the effects of the RasGRF2 KO on nicotine reinforcement.

## Materials and methods

### Animals

Male C57Bl/6N (Charles River, Germany) and male and female RasGRF2 (Fernandez-Medarde et al., 2002) KO mice and their WT littermate controls were single-housed in a temperature-controlled (21°C) environment maintained on a 12-h light-dark cycle (lights on at 6 a.m.). Food and water was available *ad libitum*. All experiments were performed in accordance with EU guidelines on the care and use of laboratory animals and were approved by the local animal care committee (Regierungspräsidium Karlsruhe, Germany). All experiments were conducted during the light phase between 8:00 and 13:00 h.

### Drugs

(-)-Nicotine ditartrate (Sigma-Aldrich, Germany) was dissolved in physiological saline (0.9% NaCl) for 0.4 mg/kg (10 ml/kg IP) administration for peripheral acute and repeated studies and 0.03 mg/kg/35 µl infusion for intravenous SA (pH to approximately 7.0 using NaOH). All doses are based on the free base weight of nicotine.

### Apparatus and procedures

#### pERK immunohistochemistry in male C57Bl/6N and RasGRF2 mice

For acute nicotine exposure, male C57Bl/6N or RasGRF2 KO and WT mice were habituated to saline injections for 4 days. The following day, mice were injected with nicotine or saline (0.4 mg/kg IP; C57Bl/6N mice, *n* = 5 per group; RasGRF2 KO and WT mice, *n* = 3–4 per group). For repeated nicotine exposure, male RasGRF2 KO and WT mice were given 10 daily injections of nicotine or saline (0.4 mg/kg IP; 4–5 per group).

Twenty minutes following the acute injection or final injection following repeated exposure, mice were anesthetized with isoflurane and transcardially perfused with phosphate-buffered saline (PBS) and 4% paraformaldehyde (PFA) in PBS. Brains were removed and placed in 4% PFA in PBS for 24 h for post-fixation, and then transferred into 20% sucrose in PBS for at least 24 h and finally into 30% sucrose in PBS until processing. Immunohistochemistry was performed following the protocol described previously (Bernardi et al., 2019; Olevska et al., 2021). Free-floating sections were rinsed in TBS and then incubated for 15 min in a quenching solution containing 3% H_2_O_2_ and 10% methanol. One hour after blocking in 5% normal goat serum and 0.1% Triton X-100, sections were incubated overnight at 4°C with anti-phospho-p44/42 MAP kinase (Thr202/Tyr204) (1:1,000, Cell Signaling Technology Cat# 4370L, RRID:AB_231511). The next day, slices were rinsed in TBS and incubated with biotinylated goat anti-rabbit IgG (1:200, Vector Laboratories Cat# BA-1000, RRID:AB_2313606) for 2 h at room temperature. Detection of the bound antibodies was carried out using a standard peroxidase-based method (ABC-kit, Vectastain, Vector Labs), followed by incubation with a DAB and H_2_O_2_ solution. Images were acquired from the dorsal and ventral striatum with a bright field microscope (Leica DM2000LED Macro/micro imaging system) at ×20 magnification. Neuronal quantification was carried out using ImageJ software. The total number of pERK1/2-positive cells was counted in the dorsal and ventral striatum in 2–4 rostral sections per mouse. The pERK count from one animal in the acute experiment was identified as a significant outlier in the dorsal striatum using Grubbs’ test and thus the animal was removed from all further analyses.

#### Nicotine self-administration in male and female RasGRF2 mice

Nicotine SA was assessed in 8 operant chambers (Med Associates, United States) housed in light- and sound-attenuating cubicles. Each chamber (24.1 cm × 20.3 cm × 18.4 cm) is equipped with two levers (left and right), a food dispenser and a drug delivery system connected via infusion pump (PHM-100, Med-Associates, United States) located outside the cubicle. Operant chambers were controlled using Med-PC IV (Med Associates, United States) software. Male and female RasGRF2 mice first underwent lever training with 14 mg sweetened food pellets (TestDiet, United States), as previously described (Bernardi and Spanagel, 2013). Following lever training, mice were implanted with an indwelling intravenous catheter (made in-house) into the jugular vein. Catheter patency was maintained with 0.15 ml heparinized saline (100 i.u./ml) containing Baytril (0.7 mg/ml) administered daily throughout the experiment. After 3 days recovery, mice underwent daily nicotine SA for 10 consecutive days. Nicotine (0.03 mg/kg/35 µl infusion) delivery was contingent upon pressing on the active lever under an FR2 schedule of reinforcement and paired with the 20s presentation of a blinking light stimulus (Conditioned Stimulus, CS), which also served as a timeout period, during which lever presses were not reinforced. The number of nicotine infusions achieved was limited to 10 and 15 infusions during sessions 1 and 2, respectively, the achievement of which resulted in the end of the session. The session continued for 1 h if these limits were not met. This limit was to ensure that excessive intake during initial access to nicotine did not become aversive. This procedure results in sustained nicotine intake across trials (personal observation from R.E. Bernardi). Days 1 and 2 are depicted in all figures, but not included in data analyses. Sessions 3–10 were 1 h in duration. Presses on the inactive lever were recorded but had no scheduled consequences. Nicotine SA was assessed in male and female RasGRF2 WT (male, *n* = 10; female, *n* = 13) and KO (male, *n* = 12; female, *n* = 11) mice.

### Statistical analysis

Statistical analyses were conducted using SPSS software (StatSoft, United States). Immunohistochemical data were conducted using two-way ANOVAs followed by Bonferroni-corrected independent samples t-tests, where indicated. All SA data were analyzed across days 3–10 using ANOVAs, as indicated, followed by Bonferroni-corrected independent samples t-tests, where indicated. Significance was set at *p* < 0.05.

## Results

### pERK is significantly increased in the striatum of male C57Bl/6N mice

**FIGURE 1:**
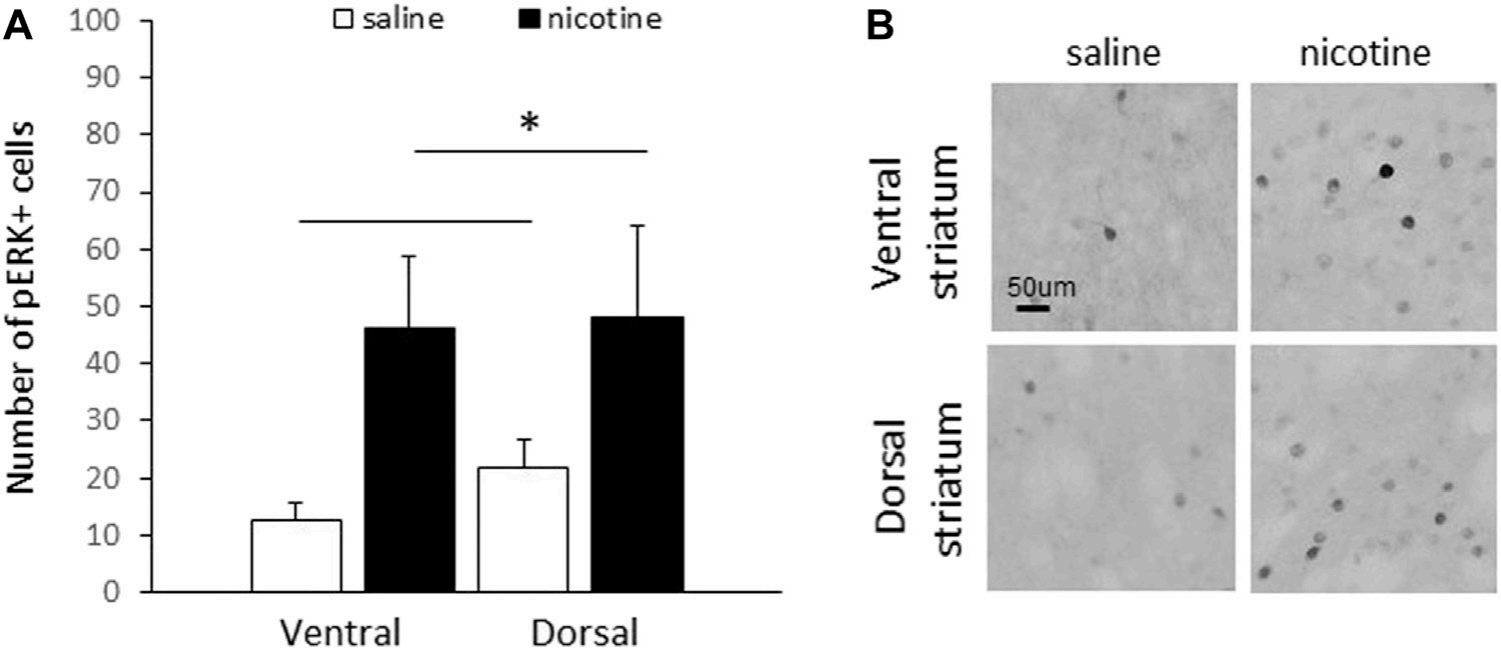
Acute nicotine increased striatal pERK in male C57Bl6/N mice. **(A)** An acute injection of nicotine (0.4 mg/kg IP) resulted in a significant increase in the number of pERK1/2-positive cells relative to saline controls in the striatum (*n* = 5/group). Data represent the mean number of pERK1/2-positive cells (±SEM) in each condition in the ventral and dorsal striatum. **(B)** Representative slices showing pERK-positive cells from the ventral and dorsal striatum of male C57Bl6N mice that received acute nicotine or saline injections. *p < 0.05, main effect of treatment (nicotine relative to saline).

Following acute nicotine injections, male C57Bl/6N mice demonstrated a significant increase in the number of pERK1/2-positive cells in response to nicotine relative to saline controls in the striatum. Figure 1A shows the mean number of pERK1/2-positive cells (±SEM) in the ventral and dorsal striatum following acute saline (*n* = 5) or nicotine (*n* = 5) administration in C57Bl/6N mice. A two-way ANOVA (region x treatment) of the number of pERK1/2-positive cells revealed a significant main effect of treatment [F (1, 16) = 8.1, *p* = 0.011], but no other significant effects [Fs < 1]. Figure 1B shows representative slices of pERK1/2-positive cells from the ventral and dorsal striatum of C57Bl6N mice administered saline or nicotine.

### pERK is significantly increased in the striatum following acute nicotine in male WT, but not RasGRF2 KO mice

**FIGURE 2:**
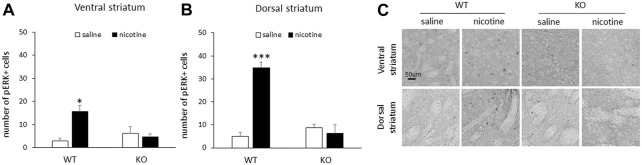
Acute nicotine failed to increase striatal pERK in male RasGRF2 KO mice. **(A)** An acute injection of nicotine (0.4 mg/kg IP) resulted in a significant increase in the number of pERK1/2-positive cells relative to saline controls in the ventral striatum of male RasGRF2 WT, but not KO mice (*n* = 3–4/group). Data represent the mean number of pERK1/2-positive cells (±SEM) in each condition. **(B)** An acute injection of nicotine (0.4 mg/kg IP) resulted in a significant increase in the number of pERK1/2-positive cells relative to saline controls in the dorsal striatum of male RasGRF2 WT, but not KO mice (*n* = 3–4/group). Data represent the mean number of pERK1/2-positive cells (±SEM) in each condition. **(C)** Representative slices showing pERK-positive cells from the ventral and dorsal striatum of male RasGRF2 WT and KO mice that received acute nicotine or saline injections. *p < 0.025; ***p < 0.0005.

Following acute nicotine injections, male WT mice demonstrated an increase in the number of pERK1/2-positive cells in response to nicotine relative to saline controls in both the ventral and dorsal striatum. This increase in the number of pERK1/2-positive cells was not present in either the ventral or dorsal striatum of RasGRF2 KO mice (*n* = 3–4/group). Figure 2A shows the mean number of pERK1/2-positive cells (±SEM) in the ventral striatum in RasGRF2 KO and WT mice sacrificed following an acute injection of saline or nicotine. A two-way ANOVA (genotype × treatment) of the number of pERK1/2-positive cells revealed significant main effects of treatment [F (1, 9) = 6.1, *p* = 0.035] and a significant genotype × treatment interaction [F (1, 9) = 9.9, *p* = 0.012], but no effect of genotype [F (1, 9) = 2.7, *p* = 0.14]. Independent samples t-tests revealed a significant increase in the number of pERK1/2-positive cells in WT mice administered nicotine relative to saline-treated mice [t (5) = 3.8, *p* = 0.012; Bonferroni-corrected *α*/2 = 0.025] that was not present in KO mice [t (4) = 0.43, *p* = 0.69; Bonferroni-corrected *α*/2 = 0.025]. Figure 2B shows the mean number of pERK1/2-positive cells (±SEM) in the dorsal striatum in RasGRF2 KO and WT mice sacrificed following an acute injection of saline or nicotine. A two-way ANOVA (genotype x treatment) of the number of pERK1/2-positive cells revealed significant main effects of genotype [F (1, 9) = 23.2, *p* = 0.001] and treatment [F (1, 9) = 28.6, *p* < 0.0005], and a genotype × treatment interaction [F (1, 9) = 39.0, *p* < 0.0005]. Independent samples t-tests revealed a significant increase in the number of pERK1/2-positive cells in WT mice administered nicotine relative to saline-treated mice [t (5) = 9.1, *p* < 0.0005; Bonferroni-corrected *α*/2 = 0.025] that was not present in KO mice [t (4) = 0.57, *p* = 0.60; Bonferroni-corrected *α*/2 = 0.025]. Figure 2C shows representative slices of pERK-positive cells from the ventral and dorsal striatum of RasGRF2 KO and WT mice administered saline or nicotine.

### pERK is significantly increased in the striatum following repeated nicotine in male WT, but not RasGRF2 KO mice

**FIGURE 3:**
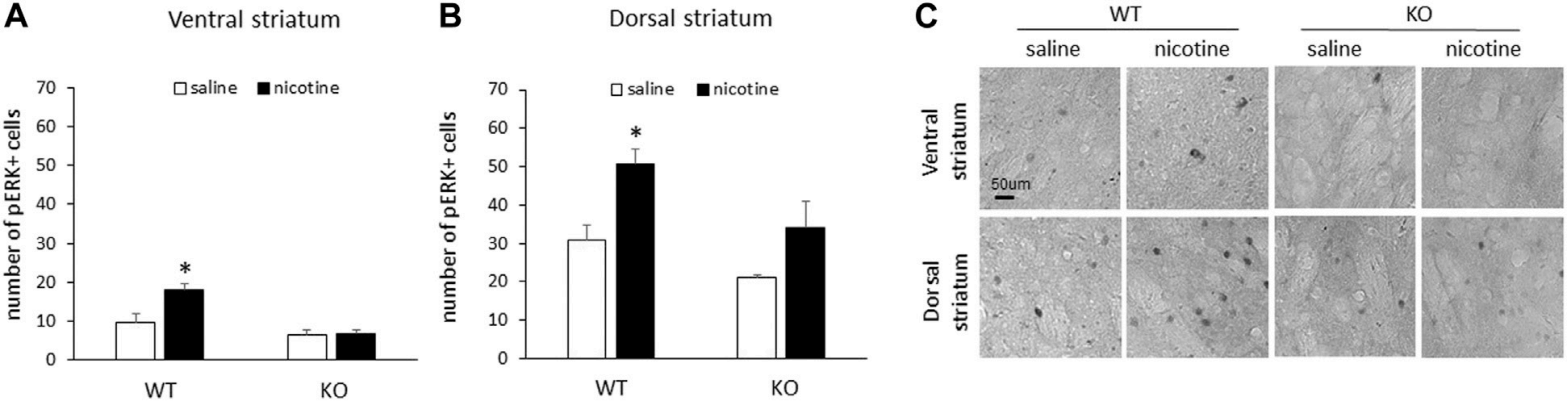
Repeated nicotine failed to increase striatal pERK in male RasGRF2 KO mice. **(A)** 10 days of nicotine (0.4 mg/kg IP) injections resulted in a significant increase in the number of pERK1/2-positive cells relative to saline controls in the ventral striatum of male RasGRF2 WT, but not KO mice (*n* = 4–5/group). Data represent the mean number of pERK1/2-positive cells (±SEM) in each condition. **(B)** 10 days of nicotine (0.4 mg/kg IP) injections resulted in a significant increase in the number of pERK1/2-positive cells relative to saline controls in the dorsal striatum of male RasGRF2 WT, but not KO mice (*n* = 4–5/group). Data represent the mean number of pERK1/2-positive cells (±SEM) in each condition. **(C)** Representative slices showing pERK-positive cells from the ventral and dorsal striatum of RasGRF2 WT and KO mice that received acute nicotine or saline injections. *p < 0.025.

Following repeated nicotine exposure, male WT mice demonstrated an increase in the number of pERK1/2-positive cells in response to nicotine relative to saline controls in both the ventral and dorsal striatum. This increase in the number of pERK1/2-positive cells was not present in either the ventral or dorsal striatum of RasGRF2 KO mice (*n* = 4–5/group). Figure 3A shows the mean number of pERK1/2-positive cells (±SEM) in the ventral striatum in RasGRF2 KO and WT mice sacrificed on day 10 following repeated injections. A two-way ANOVA (genotype × treatment) of the number of pERK1/2-positive cells revealed significant main effects of genotype [F (1, 14) = 25.0, *p* < 0.0005] and treatment [F (1, 14) = 8.4, *p* = 0.012], and a significant genotype × treatment interaction [F (1, 14) = 7.5, *p* = 0.016]. Independent samples t-tests revealed a significant increase in the number of pERK1/2-positive cells in WT mice administered nicotine relative to saline-treated mice [t (7) = 3.3, *p* = 0.013; Bonferroni-corrected *α*/2 = 0.025] that was not present in KO mice [t (7) = 0.15, *p* = 0.88; Bonferroni-corrected *α*/2 = 0.025]. Figure 3B shows the mean number of pERK1/2-positive cells (±SEM) in the dorsal striatum in RasGRF2 KO and WT mice sacrificed on day 10 following repeated injections. A two-way ANOVA (genotype × treatment) of the number of pERK1/2-positive cells revealed significant main effects of genotype [F (1, 14) = 7.8, *p* = 0.014] and treatment [F (1, 14) = 12.3, *p* = 0.003], but no significant genotype × treatment interaction [F (1, 14) = 0.51, *p* = 0.49]. Independent samples t-tests revealed a significant increase in the number of pERK1/2-positive cells in WT mice administered nicotine relative to saline-treated mice [t (7) = 3.6, *p* = 0.008; Bonferroni-corrected *α*/2 = 0.025] that was not present in KO mice [t (7) = 1.7, *p* = 0.12; Bonferroni-corrected *α*/2 = 0.025]. Figure 3C shows representative slices of pERK-positive cells from the ventral and dorsal striatum of RasGRF2 KO and WT mice administered repeated saline or nicotine.

### Nicotine self-administration is increased in male and female RasGRF2 KO mice

**FIGURE 4:**
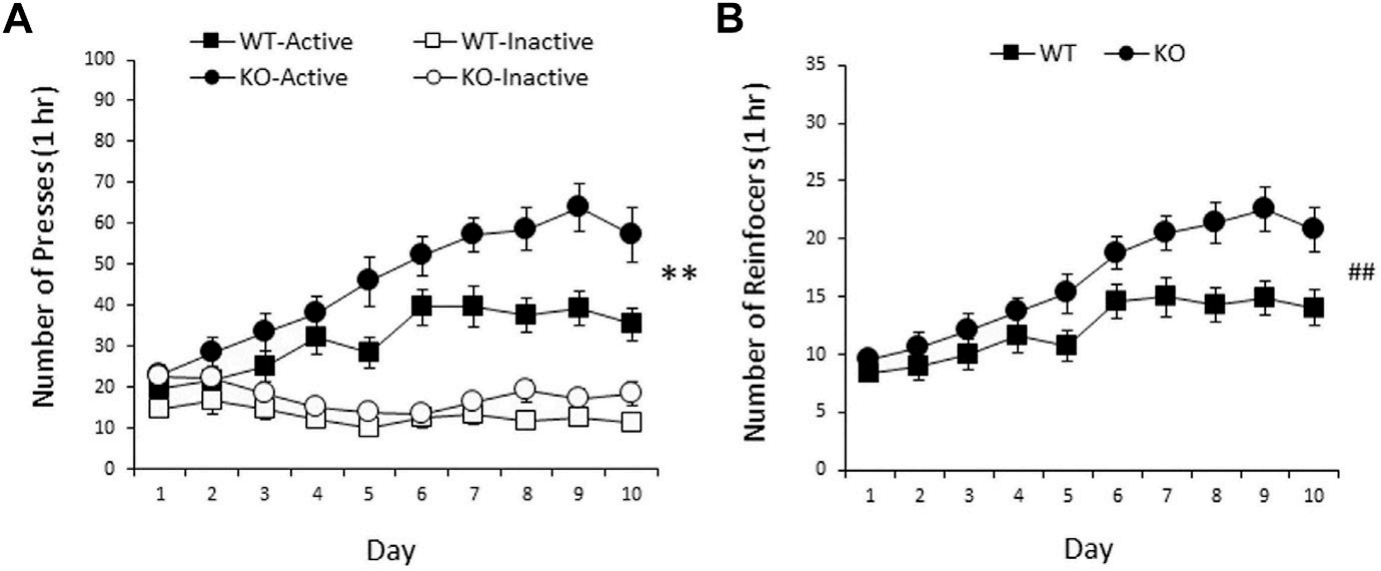
Nicotine SA in male and female RasGRF2 KO mice and WT controls. **(A)** RasGRF2 KO mice demonstrated an increase in responding on the nicotine-associated lever relative to WT controls (*n* = 23/group). Data represent mean number of presses (±SEM) on the active and inactive levers during 10 daily 1 h sessions of nicotine SA (0.03 mg/kg/35 µl infusion). **(B)** RasGRF2 KO mice demonstrated an increase in the number of nicotine reinforcers achieved relative to WT controls. Data represent mean number of nicotine reinforcers (±SEM) achieved during 10 daily 1 h sessions of nicotine SA (0.03 mg/kg/35 µl infusion). **p < 0.005; ^##^ *p* < 0.005, main effect of genotype.

RasGRF2 KO and WT mice differed in nicotine SA, with RasGRF2 KO mice demonstrating an increase in responding on the nicotine-associated lever and nicotine intake. A three-way ANOVA (sex × genotype × day) of days 3–10 revealed no significant sex effects on nicotine intake [sex: F < 1; sex × genotype: F < 1; sex × day: F (3.6, 152.7) = 1.5, *p* = 0.22; sex × genotype × day: F (3.6, 152.7) = 2.0, *p* = 0.10]. Thus, the data for male and female mice were combined for subsequent analyses (*n* = 23 mice/group). Figure 4A shows the mean (±SEM) responding on the active and inactive levers during 10 daily sessions of nicotine SA in RasGRF2 KO mice and WT controls. A three-way ANOVA of lever responses during 1 h sessions on days 3–10 (lever × day × genotype) revealed a significant main effect of lever [F (1, 44) = 145.7, *p* < 0.0005], indicating a distinction between the active and inactive levers, and a significant effect of day [F (4.1, 181.1) = 6.8, *p* < 0.0005] and day × lever interaction [F (4.0, 173.9) = 13.1, *p* < 0.0005]. Most importantly, there was a significant main effect of genotype [F (1, 44) = 11.3, *p* = 0.002], as well as a significant lever × genotype interaction [F (1, 44) = 6.5, *p* = 0.015], indicating a difference in responding between KO and WT controls over the final 8 days of nicotine SA. No other effects reached significance [day × genotype: F (4.1, 181.1) = 1.8, *p* = 0.14; lever × day × genotype: F (4.0, 173.9) = 1.6, *p* = 0.18]. Independent samples t-tests confirmed that RasGRF2 KO mice responded significantly more on the active lever than WT mice [t (44) = 3.3, *p* = 0.002; Bonferroni-corrected *α* = 0.025] across days 3–10, but the two groups did not differ on inactive lever pressing [t (44) = 1.9, *p* = 0.066; Bonferroni-corrected *α* = 0.025], indicating a selective increase in responding on the nicotine-associated lever by RasGRF2 KO mice relative to WT controls. Figure 4B shows the mean (±SEM) number of nicotine infusions received during 10 days of nicotine SA in RasGRF2 KO mice and WT controls. A two-way ANOVA of nicotine reinforcers during 1 h sessions on days 3–10 (day × genotype) revealed significant main effects of genotype [F (1, 44) = 9.5, *p* = 0.004], and day [F (3.7, 163.4) = 13.2, *p* < 0.0005], but no day × genotype interaction [F (3.7, 163.4) = 1.8, *p* = 0.13]. These findings indicate a significant increase in nicotine intake in RasGRF2 KO mice relative to WT controls over the final 8 days of nicotine SA.

## Discussion

Here we sought to extend our previous findings of the contribution of RasGRF2 to cocaine SA using nicotine SA in RasGRF2 KO. In the current study we used the peripheral administration of nicotine to demonstrate that nicotine reliably increases pERK1/2 in the striatum following both acute and repeated injections, an effect not present in RasGRF2 KO mice. Furthermore, RasGRF2 KO mice demonstrated an increase in nicotine SA relative to WT mice. These data suggest that RasGRF2 is involved in the reinforcing properties of nicotine likely *via* its regulation of activity of the Ras-ERK pathway. Furthermore, these data and those of previous studies of alcohol and cocaine SA further implicate the Ras-ERK signaling pathway as a common mediator of drug reinforcement.

Our demonstration of an increase in the number of pERK1/2-positive cells in the striatum of C57Bl/6N mice following acute nicotine as compared to saline administration is consistent with previous reports that showed acute nicotine-induced increases in ERK1/2 in the striatum using an identical dose of nicotine (Valjent et al., 2004; Valjent et al., 2005). Furthermore, although our focus here was on striatal regions, due to the fact that both the ventral and dorsal striatum have been demonstrated to play a role in the rewarding/reinforcing properties of drugs of abuse, including nicotine (Pascual et al., 2009; Veeneman et al., 2012; Veeneman et al., 2015), acute nicotine has also previously been demonstrated to increase pERK1/2 in several other brain areas that are important for drug-mediated neuroadaptations and consequent drug-seeking responses, such as the PFC, central nucleus of the amygdala, and bed nucleus of the stria terminalis (Valjent et al., 2004; Koob and Volkow, 2010).

RasGRF2 WT mice demonstrated an acute nicotine-induced increase in pERK1/2 in the ventral striatum, consistent with our findings above and previous reports (Valjent et al., 2004; Valjent et al., 2005). RasGRF2 KO mice failed to show this increase. In addition, nicotine also induced an increase in pERK1/2-positive cells relative to saline-treated controls in the dorsal striatum in RasGRF2 WT mice, also absent in RasGRF2 KO mice. These findings suggest that the attenuation of an acute nicotine-induced increase in pERK1/2 is at least partly mediated through the Ras-ERK pathway via RasGRF2. These findings are also consistent with a general role of striatal Ras-ERK signaling in the acute effects of a variety of drugs of abuse. For example, we previously demonstrated that an injection of acute cocaine in mice resulted in an increase in pERK1/2 that was impaired when preceded by administration of the mitogen-activated protein kinase (MEK; an upstream regulator of ERK) inhibitor PD325901 (Papale et al., 2016; see also Valjent et al., 2000). Similar results demonstrating MEK inhibitor attenuation of drug-mediated increases in pERK in striatal regions in rodents have been found with other drugs of abuse, including amphetamine (Shi and McGinty, 2006), morphine (Mouledous et al., 2007), and alcohol (R.E. Bernardi, unpublished results), suggesting a critical role of the Ras-ERK signaling pathway in the cellular response to drugs of abuse.

Following 10 days of peripheral nicotine administration, RasGRF2 WT mice demonstrated a significant increase in pERK1/2 in both the ventral and dorsal striatum, similar to our findings above following acute injections. RasGRF2 KO mice failed to show this increase in the ventral or dorsal striatum, although in the dorsal striatum there was a modest increase in pERK1/2 that failed to reach significance, due to a single animal showing a high pERK1/2 expression following nicotine administration. These findings again suggest that nicotine produces reliable increases in pERK1/2 in brain areas associated with the effects of drugs of abuse, and implicate RasGRF2 and the Ras-ERK pathway as a likely mediator of these effects. A previous study found no differences in pERK1 or pERK2 in the NAc following chronic administration, though differences in hypothesis and subsequent study design (nicotine in drinking water) likely explain this contrasting result (Brunzell et al., 2003). In fact, Yang et al. (2021) recently demonstrated that 7 days of peripheral nicotine injections in rats resulted in an increase in pERK1 and pERK2 in the NAc relative to saline controls. Furthermore, a loss of nicotine-induced increases in pERK has been correlated with impairments in nicotine CPP (Wilar et al., 2019; Jia et al., 2021).

Nicotine SA in RasGRF2 KO mice resulted in an increase in SA relative to WT mice. These data replicate our previous findings of cocaine SA in RasGRF2 KO and WT mice (Bernardi et al., 2019). Given our pERK1/2 findings, this result likely represents a compensatory increase in intake resulting from decreased nicotine reward resulting from the RasGRF2 KO—a decrease in the magnitude of the nicotine reinforcer that requires an increase in intake to achieve a similar putative subjective effect—similar to attenuation of reward via decreases in dose or pharmacological means that results in compensatory increases in intake (De Wit and Wise, 1977; Ettenberg et al., 1982; Caine and Koob, 1994; Thomsen and Caine, 2006; Faccidomo et al., 2009). For example, Faccidomo et al. (2009) demonstrated that the MEK inhibitor SL327 administered prior to daily sessions increased alcohol SA (Faccidomo et al., 2009), a result also interpreted as an increase in intake to compensate for decreased alcohol reward. These findings are also consistent with a previous result showing a loss in alcohol-induced dopamine increase in the NAc and dorsal striatum in RasGRF2 KO mice relative to WT controls, resulting in decreased alcohol reinforcement (Stacey et al., 2012). Thus, RasGRF2 likely plays a general modulatory role in drug-mediated behaviors.

In the SA study outlined here, both male and female RasGRF2 KO and WT mice were used to identify potential sex-dependent differences in nicotine SA and its mediation by RasGRF2. Previous studies of nicotine SA in rodents have demonstrated either increased intake in males, no differences in intake between males and females, or higher intake in females relative to males (Donny et al., 2000; Feltenstein et al., 2012; Bernardi et al., 2016; Flores et al., 2019; Chen et al., 2020; Chellian et al., 2021; Leyrer-Jackson et al., 2021). Here we found no sex-dependent differences in nicotine intake in WT mice; however, we did not measure other specific aspects of nicotine SA that may differ among males and females, such as motivation, extended-access procedures, reinstatement, and treatment effectiveness, which are likely relevant to clinical outcomes in humans. We also found no sex-dependent difference in the alteration in nicotine intake as a function of the RasGRF2 KO. We do not know of any previous rodent study that has examined the effect of manipulation of components of the Ras-ERK pathway on measures of drug reinforcement in both males and females, although one previous cocaine conditioned place preference study demonstrated that following testing, pERK levels in the NAc and preference scores did not differ between male and female rats (Nygard et al., 2013). Thus, RasGRF2 mediation of nicotine SA is likely sex-independent.

Previous studies have implicated ERK as a coincident detector of D1R-mediated signaling and NMDA receptor activation in the striatum critical to the regulation of drug-dependent synaptic plasticity in the striatum (Zhang et al., 2004; Valjent et al., 2005; Jiao et al., 2007; Bertran-Gonzalez et al., 2008; Fasano et al., 2009; Ren et al., 2010; Morella et al., 2020). For example, both NMDA and D1 antagonists have been shown to block ERK phosphorylation in the striatum in response to a variety of drugs of abuse (Valjent et al., 2000; Valjent et al., 2004; Valjent et al., 2005). The striatal increase in pERK induced by drugs of abuse was also attenuated in D1 KO mice relative to controls (Valjent et al., 2005). Furthermore, previous studies have implicated RasGRF2 in the synaptic plasticity associated with both NMDA- (Tian et al., 2004; Jin and Feig, 2010; Jin et al., 2014) and D1-mediated stimulation (Stacey et al., 2012). Thus, in terms of nicotine and other drugs of abuse, RasGRF2 likely mediates drug reward via the activity of ERK in response to D1- and NMDA-mediated signaling.

It should be noted that although our results here are consistent with those of our previous cocaine paper using RasGRF2 KO mice (Bernardi et al., 2019), in that nicotine SA was increased in RasGRF2 KO mice relative to WT controls and KO mice failed to show the increase in pERK1/2 in response to nicotine demonstrated in WT mice, we used peripheral injections of nicotine for our acute and chronic immunohistochemical findings. Although the dose of nicotine for IP injection was virtually identical to the amount achieved via daily SA (based on mean intake), our peripheral nicotine injections do not represent a measure of reward with which to directly link changes in pERK1/2 expression. Thus it is possible that changes in nicotine-induced pERK1/2 expression in the striatum do not reflect alterations in reinforcement associated with nicotine SA. However, previous work has implicated alterations in pERK1/2 expression with changes in nicotine reward (Wilar et al., 2019; Jia et al., 2021). For example, Wilar et al. (2019) demonstrated that pERK1/2 was increased following nicotine CPP in WT mice relative to vehicle-treated animals, but this increase in pERK1/2 expression and subsequent nicotine CPP was absent in Dopamine D2 receptor KO mice. Furthermore, in our previous cocaine paper, in which we demonstrated a direct link between cocaine reinforcement and the expression of pERK1/2 (Bernardi et al., 2019; see also Papale et al., 2016), we also demonstrated that the behavioral effect of pERK1/2 inhibition on SA was dependent on whether the manipulation was peripheral/global or site-specific. In other words, both the RasGRF2 KO and peripheral administration of the MEK inhibitor PD325901 resulted in an increase in cocaine intake, while viral-mediated RasGRF2 knockdown and MEK inhibition in the NAc both resulted in a decrease in cocaine SA. Further studies will need to determine the precise mechanisms by which these global and site-specific findings produce differing effects on behavior but an identical inhibition of the expression of pERK1/2, and whether similar findings occur with nicotine SA. Nonetheless, these findings suggest that alterations in pERK may mediate drug intake.

In summary, we demonstrated that RasGRF2 is involved in nicotine reinforcement associated with operant SA, likely by regulating the activity of ERK. In combination with previous findings, these studies further implicate a common mechanism for the Ras-ERK pathway in the effects of drugs of abuse.

## Data Availability

The raw data supporting the conclusion of this article will be made available by the authors, without undue reservation.
